# British Dental Association

**Published:** 1887-10

**Authors:** 


ARTICLE II.

### BRITISH DENTAL ASSOCIATION.

The annual general meeting of the British Dental Association was held in the Hall of the Faculty of Physicians and Surgeons, Glasgow, on the 18th, 19th and 20th ult. Sir Edwin Saunders, F. R. C. S., President, occupied the chair, and there was an attendance of about 120 members.

The President, in opening the meeting for Association business, announced the resignation of Sir John Tomes from the office of President of the Representative Board of the Association. He was afraid they must accept this resignation as final. Sir John had been induced last year to withdraw his resignation for a year, but his inability to attend the meetings had, as he explained, rendered him unable to hold out a prospect of any longer being President. But, at the same time, he was quite ready on all occasions to give the Association his valuable advice, which they would esteem in all cases of difficulty, because no man, perhaps, was better able to advise when any questions of difficulty arose, from his long observation and experience of everything connected with the institution, and from the singleness of purpose with which he had, at all times, devoted himself to the interests of the Association.

The letter of resignation and a valedictory address by Sir John Tomes to the Representative Board having been read, the President moved, and it was resolved—"That this meeting accept with great reluctance the resignation of Sir John Tomes as President of the Representative Board of the British Dental Association, and desires to record its deep sense of the value of his services in the cause of dental reform and professional organization, and especially of his vigilance during a long period of impending legislation in medical matters which has resulted in securing a full recognition and place for dental surgery in the recent and probably final Medical Amendment Act of 1886."

It was afterwards agreed that the vacancy caused by the retirement of Sir John Tomes should be filled up by the appointment of Mr. Smith Turner, who has hitherto acted as Vice-president of the Representative Board; and Mr. Felix Weiss was appointed Vice-president in room of Mr. Smith Turner.

Mr. F. Canton, Treasurer *pro tem.*, reported that the balance in bank at the credit of the Association was £589 16s. 2d.

Mr. Morton Smale, Secretary, stated that the total number on the roll of the Association was 645, being an increase of 38 over last year.

It was agreed that the next meeting should be held in Dublin on the 23rd, 24th and 25th August, 1888.

Mr. Daniel Corbett, Dublin, President of the Irish Branch, was elected President-elect; and Mr. F. Canton was appointed Treasurer in room of Mr. James Parkinson, who had been obliged to resign on account of ill-health.

Sir Edwin Saunders, in his valedictory address, said the question of greatest moment to them was what had been the effect of the past year's operations of the Association; what was the record of its work, and to what extent had it fulfilled its purpose? Had it satisfied or fallen short of the expectations of the profession in whose interests it was established? He could not help thinking that the reports to which they had listened gave a very satisfactory answer to these questions. This phenomenal year, which had witnessed an unparalleled outburst of patriotism and loyalty, this jubilee year, when they were alternately filled with amazement at the fervour of attachment of all classes and conditions to the personality of our Sovereign, and at the benignity of nature in the unwonted affluence of sunshine with which our beloved fatherland had been favoured —would be remembered by the members of the Association as having been signalized by the formation of the Irish branch. This gratifying event, the consummation of which was greatly due to the prompt action of Mr. Smith Turner, Vice-chairman of the Representative Board, whose energy and unselfish devotion to duty had been displayed on many similar occasions, would, he felt sure, be regarded as a matter of sincere congratulation by the Association, at once as an undoubted accession of strength not only by the augmented number of members, but by the social position of the gentleman by whom their profession had been so well represented in the sister isle. For them he hoped the affiliation would be productive of freer intercourse, warmer friendships, more liberal judgments of each other, greater dignity and prestige, with a higher estimation of the profession on the part of the general and medical public, and all the social gifts and privileges which spring from united action for the common good.

The two great engines for raising the dental profession were an educated public opinion and organized instruction in dental science; and these were working steadily and surely for this end. If they looked back only some twenty years they could not fail to be struck with the real and substantial advance which had been made not only in the development and resources of the art, but in the social and educational status of those now entering its ranks. And it was in this direction that would be found the true and efficient means of at once freeing it from opprobrium and purging it from the contamination of the unworthy.

On the motion of Dr. John Smith, Edinburgh, seconded by Mr. Charles S. Tomes, London, the retiring President was awarded a vote of thanks for his services during the year.

Mr. J. R. Brownlie, President, then took the chair. In his opening address he said it was his pleasing duty to welcome the British Dental Association to Glasgow—to a city having associated with it memories which they must all be disposed to cherish, and memories, too, which, save for the lessons they taught, they could now afford to forget. He would remind them that they were now on what should be classic ground, in a neighbourhood possessing a special interest from having been the scene of the first essay in life of the man who had been fitly described as the father of the dental profession. In the near neighbourhood John Hunter was born, and in Glasgow he worked as a tradesman during his earlier years, before devoting himself to more congenial work, and in which he so distinguished himself. It was pleasant, he continued, to note the interest taken in them, the youngest relative, by the medical profession, as they saw it in the action of its representative institutions. Ancient seats of learning were interested in their progress, and medical schools were competing for the privilege of educating their youth.

The license in dental surgery was growing in number and in public appreciation. He knew not if it was the company Parliament decreed they should keep which had stimulated so many to take advantage of the *sine curriculo* provisions, but he knew that in this neighbourhood the possession of the license in dental surgery was increased from one within ten years anterior to the passing of the Act to 50 per cent. within ten years thereafter.

Amongst the more important results of legislation they must give a very prominent place to the British Dental Association. Formed to give effect to the spirit and the provisions of the Dentists' Act, it was yearly drawing closer professional ties. It was bringing together those who might not otherwise have met, and under conditions which could not fail to promote the art and the interests of the dentist. But it would be to misrepresent the situation to make it appear that they had as yet great occasion to rest and be thankful. A first attempt at legislation would necessarily leave room for improvement. Already certain alterations had been effected with advantage, and it might be expected that from time to time opportunities would present themselves for further adjustment of the provisions of the Act.

Of medical titles at home and dental titles abroad there was no lack, and so long as they were legitimately acquired and used the additional information and experience they represented must prove a gain to their possessor and to the credit of the profession of which he was a member. But the medallion had its obverse. The most coveted distinctions had been imitated. They had not now to complain so much of absolutely base metal as of the extent to which it was alloyed. Surely two out of so many (25) dental diplomas granted in America was too small a proportion to be found deserving a place on their dental register. Probably they ought now to congratulate themselves that the issue of really counterfeit diplomas was at an end. In that respect some improvement had been effected. It was over a dozen years since they had in Glasgow an agency for the sale of bogus diplomas—since he was favoured with a visit by the accredited agent of one of these so-called American colleges, having full authority to examine *in absentia*, and to grant any or as many titles as might be wanted. This man's agency extended to granting a degree of doctor in divinity, medicine, dentistry, and some others, and the charge was moderate, considering the outlay usually entailed in the acquisition of such honours. So flagrant an abuse could not long survive. This "college" was suppressed, and it did not appear that such honours were now to be had on the like easy terms.

They had, however, something still to hope for at the hands of their Transatlantic brethren. They were still left to contend with the varying value of American dental diplomas, nor could they be content that the title conferred should acquire by contraction a fictitious value, that it should have one meaning in America and a different meaning here.

In conclusion, he said their Association had assumed a most comprehensive title, and it had yet to make good its claim to it. The name included as a legitimate field of the Association's operations the whole of an empire on which the sun never sets. Were they to understand the term British as employed in its limited or its widest sense? His answer to that was that their sphere of action had been limited, and they might still increase within this limited sphere. But this could not be accomplished until they had taken possession of the wider area; and not till the Association was represented by branches wherever dentists were in numbers sufficient to combine for the purpose could they claim to be, in the fullest acceptance of the term, the "British Dental Association."

The reading of papers was next proceeded with, the first being on

THE HASTIE WATER MOTOR, or the water pressure to be obtained in all towns in Great Britain and Ireland of above 10,000 inhabitants, with remarks in referance to the use of the Hastie motor in workrooms and surgeries, by W. Campbell, L. D. S. Eng., Dundee. This was read by the Secretary, Mr. Smale. In the course of the paper, Mr. Campbell gave his experience in working the Hastie motor in his surgery. The machine has proved very serviceable, but was still capable of improvement. When a sufficiently perfect machine was obtained, motors, whether Hastie's or some other inventor's would, he believed, be used by all dental surgeons, and would be found in all dental work-rooms. For the proper working of the machine it was necessary to have a continuous and sufficient pressure of water. The paper closed with a list of the various water pressures to be obtained in the chief cities and towns in the United Kingdom.

CONTINUOUS GUM WORK WITH ANY FORM AND MAKE OF TOOTH, by James Cumming, L. D. S. Glasgow. Although they had made great strides of late in both surgical and mechanical dentistry, there seemed to be yet room for improvement. It was to what he thought was an improvement in a process in the latter branch of their profession that he would call their attention. Most of them had seen, and some had tried, the Verrier process of producing continuous gum facings. Probably all would admit that there was considerable difficulty and uncertainty in producing a satisfactory piece of work of this kind by the present process. First of all, a particular kind of tooth must be used; secondly, when the work was finished, there was considerable warpage, sometimes entirely spoiling articulation; thirdly, the teeth get fused and made unsightly on the surface from being exposed to the great heat required to fuse the gum-facing; and, fourthly, there was the tendency of the facing to crack, even when the piece was being worn in the mouth, and without any apparent cause. The improvements he had been working out would certainly do away with the first three defects mentioned, and, to a great extent, if not entirely, with the fourth. In his process, which he explained at some length, the advantages were that a broken tooth could be replaced easily, and any form of tooth could be used. When all this could be done without the use of pure gold as a solder, and the teeth wholly untouched by the fire, they would surely agree with him that it should be the coming process of continuous gum facing.

Specimens of the work were passed round for inspection, and an interesting discussion followed.

Mr. Gordon Jones, L. D. S. I., London, next read a paper on

### A MORE EFFICIENT METHCD OF CONFERRING DENTAL APPOINTMENTS.

He protested against the general rule in provincial towns of conferring dental appointments in connection with institutions on gentlemen having either the most influence or largest practice, thus asking them to perform duties which they had not the time to discharge conscientiously.

### THE MECHANICAL TRAINING OF DENTAL STUDENTS.

George Cunningham, B. A., D. M. D., L. D. S. Eng., Cambridge, read a paper on the above.

There might perhaps exist, he said, certain laboratories where complete and efficient mechanical training might be obtained, but he feared they were few and far between. If they put aside all questions of self-interest which they might have as instructors, they must admit that the present system of pupilage had its drawbacks as well as its advantages. His main proposal was that the whole of the time of the dental pupil should not be exclusively passed in the dental laboratory, but that, where suitable institutions existed, a tangible part of each day, or even alternate days, should be spent in attendance at some practical and allied courses of manual technology or in a practical school of mechanics.

The conditions and the actual courses followed must vary according to the various opportunities afforded in the different parts of the kingdom. Mr. Morton Smale, he said, held that a practical examination in the mechanical department should form a part of the ordeal to be passed before the student obtained his coveted diploma. He trusted the Association would cordially support such a proposition, as thereby it would be the sooner carried into force. That proposition, however, seemed to him only another very strong reason why the dental student should be furnished with an opportunity of remedying the possible defects and deficiencies of his private pupilage by the optional and public course of one year in a practical technical school. Some such arrangement as he had just indicated would combine the advantages of the school system with that of the present form of pupilage, and certainly would do much to promote the efficiency of L. D. S. diploma, which, after all, must ever be the essential qualification for a thorough dental practitioner.

A long discussion followed the reading of the paper, in which Messrs. Smale, Pearsol, Gaddes, Smith, Woodburn, Wilson, Fothergill, Turner, Brunton and Stack took part.

In the evening the President and Mrs. Brownlie held a reception of the members of the Association and their friends in St. Andrew's Hall, music and dancing adding to the enjoyment of the company.

FRIDAY, AUGUST 19th.

The business of the second day began by a meeting of the Benevolent Fund, the President, Mr. J. R. Brownlie, in the chair.

Sir Edwin Saunders, F. R. C. S., submitted the Treasurer's report, which showed that the income during the past year amounted to £369 6s. 4d. Of this sum £81 5s. 4d. was received from donations and £288 1s. from subscriptions, which, with £152 8s. 11d. in bank at the commencement of the year, made a grand total of £521 15s. 3d. There was expended during the year in benevolent allowances £180 11s. 10d., being £31 more than last year. At the present time the money in bank amounted to £210 13s. 4d., and on hand £24 15s. 1d. The income was £91 13s. 5d. greater than the previous year. The invested capital now reached £863 5s., and last year it yielded an income of £20 4s. It must be most satisfactory to the subscribers to the fund to learn that the working expenditure for the year amounted to only £5 5s.

Mr. S. J. Hutchinson read the Secretary's report. It stated that the general business brought before the Committee during the year had been on the increase compared with past years. The number of children who were being educated at the expense of the fund was eleven.

THE VALUE OF ANTISEPTICS IN DENTAL SURGERY, by E. Lloyd Williams, M. R. C. S., L. R. C. P., L. S. A., L. D. S., Eng., London.

He made no apology for introducing this subject before the Association, because he was convinced of its importance, not only in its purely scientific aspects, but more particularly in its practical bearing upon the every-day work of modern dentistry; and to the latter consideration he intended addressing himself exclusively. It would be his endeavour to touch upon the broad principles which should rule over their practice, and to suggest a few special points of treatment which had been found valuable, rather than to formulate any hard and fast laws or dogmatise upon pet theories and particular methods.

The debt of modern surgery to Sir Joseph Lister was difficult to gauge; but they were apt to lose sight of the fact that they owed less to what was known as strict

"Listerism" than to the great doctrine of cleanliness which underlay all the teaching of that eminent surgeon. The great principle which regulated all operations and which had been the very foundation of success was absolute surgical cleanliness. He feared that, as dental surgeons, they had even yet scarcely grasped the importance of that principle.

It was true that their sphere of surgical work was limited, and that they were concerned for the most part with hard tissues, which they looked upon as being comparatively indifferent to external conditions; but they must not forget that even in the simplest operation they had to do with living material, not to speak of those branches of their work where they were brought face to face with ordinary surgical conditions, and when the result of their treatment was wrapped up with the constitutional welfare or woe of their parents. Nature stood more insults in the mouth than in any other part of the body, and her long-suffering had admitted of much reckless treatment with comparative impunity. There was probably no operation on any part of the human body commensurate with that of wrenching a tooth from its bony socket where the repair was so rapid and the after effects so slight. If this were not so they should probably approach the mouth with a far greater degree of caution, and should employ every possible antiseptic precaution for procuring a minimum of local and constitutional disturbance. But it yet remained to be proved how far they could improve upon present results by being more cautious, and he felt confident that in special cases antisepticism must lead to more brilliant results if carried out with thoroughness.

There was another side to the question which must not be lost sight of. It was well for them that there were so few recorded cases of inoculation from the mouth, but that was no reason why they should employ dirty instruments. Cleanliness, then, should be an elementary but essential principle of their practice. All their instruments should be not only clean, but, where at all probable to come in contact with the soft tissues, absolutely aseptic.

There were other ways in which antiseptics might prove useful in dental work. The commonest operation which they had to perform was the preparation of cavities for the insertion of fillings, and there was always a septic condition of the tissue to be contended with. In difficult cavities these disinfectant must be of value. Speaking of the power of absolute dryness to protect susceptible bodies from putrefaction, he said he believed the use of the hot-air syringe was one of the operative reforms which was most to be desired. The air itself could be rendered aseptic by dropping into the rubber bulb of the syringe a few droys of eugenol or eucalyptus oil. As a further precaution, any unsound dentine left in the immediate neighbourhood of the pulp, having been thoroughly dried, should be painted with an antiseptic varnish and then firmly dried with a current of warm air. Mr. Williams concluded with a reference to the filling of dead teeth and the advisability of rendering even temporary fillings antiseptic.

Mr. A. Kirby, L. D. S., Eng., Bedford, read an interesting paper on

THE APPLICATION OF ELECTRICITY TO DENTAL USES.

THE DENTAL ASPECT OF PUBLIC HEALTH, by George Cunningham, B. A., D. M. D., L. D. S. Eng., Cambridge.

The efficient treatment of so great a question must no longer be left in the hands of a few, no matter how energetic they might be, and his object was to suggest some "plan of campaign" which, after due discussion and approval, would receive the weight and influence with which the corporate and united action of their Association could imbue it. Every one recognised the fact that the diseases with which the dental practitioner dealt did not directly contribute to the death-rate, and that they were neither infectious nor contagious. Hence, doubtless, arose the utter indifference of the community with regard to them; nor would that indifference be removed until the contributory and indirect influence which they had on the death-rate was better understood.

The fact of State medicine being possible marked an epoch in which some sanitary rules received a general consent, and indicated an advancing civilisation. Was it too much to demand that State medicine should include State dentistry? He thought not. It was both the duty and the interest of the State to protect the helpless child from the results of dental as well as other disease, whether arising from the ignorance, the neglect, or the incapacity of the parents to provide the necessary treatment. In this consideration the very important fact should be recognised that from a lack of attention in the period of their youth men and women of the future were allowed to grow up with the inevitable certainty of future suffering, a large proportion of whom could only be radically treated between the ages of from ten to fifteen years. This naturally brought them to the consideration of Mr. Fisher's contention for the compulsory attention to the teeth of school children; and no intelligent reader, whether professional or otherwise, could but admit that he had succeeded in proving his case. Some would be inclined, perhaps, to scout the proposition of compulsory attention as visionary and Utopian, while others, admitting the advantages of such a scheme, might think they disposed of the question by stamping it as rank socialism. Again, many hearty sympathizers with this great project were at once taken aback by the economic difficulties of its institution. It was no use disguising the fact that this was a most serious aspect of the question. Every one would be quick to recognize the increased charge upon expenditure, but comparatively few had the power that they possessed of realizing that it would be rather a mere transfer of liability already incurred in some other way, if not absolute saving of expenditure in other directions. The question might indeed be postponed for a time but a sense of patriotism should urge them on to be the leaders rather than followers in a movement which time itself would hurry on.

Without referring to the action which some of the American dental societies had taken in promoting attention to the teeth of school children—in France, where no powerful association such as theirs existed, measures had been taken for the inspection and care of the teeth of children in the schools of Paris—he would ask them for a minute to consider how this important matter affected the services. There could be no doubt whatever that the Army Medical Department recognized the advisability and the necessity of remedial treatment other than that by mere extraction. If this were not the case, no provision would have been made for the supply of tooth-stopping and scaling instruments. The nature of the provision, however, was so absurd, that they were doing a service alike to the soldier, the army surgeon, and to the already heavily-burdened taxpayer in calling attention to the fact. Who until the other day ever supposed that gold foil was provided for the filling of the soldiers' teeth; but did any army man ever know of the gold foil reaching the mouth of Tommy Atkins? In these days of Government inquiry, it might not be uninteresting to find out something about this misapplication of the nation's funds, for as experts they recognized the utter impossibility of properly manipulating not only the gold foil, but the amalgam and gutta-percha also provided, with the absurd equipment of three scalers and stoppers, and three excavators and roseheads!

Nor was that all. Not only were the materials provided absurd, and the equipment utterly inadequate, but the army regulations only provided for one such case being kept at headquarters of each military district. The least intelligent inhabitant of Glasgow would see the absurdity of the provision when he knew that the whole of Scotland constituted but one military district. But even were the materials, the instruments, and the distribution of the equipment all that they could desire, what was the use of it all if the army surgeon had had no training in necessary manipulative skill. He could not help feeling that, now the attention of the department had been called to this very important question, they should recognize and put in force the power they undoubtedly possessed of requiring all the army medical candidates to produce evidence of having received at least an elementary training in dental surgery. He concluded by submitting the following resolution:—

"That this meeting is of opinion (1) that wherever the State provides medical services dental services should be provided for as an essential part of such medical provision;

"(2) That, having regard to the great importance of securing competent attention to the teeth of the army and navy, the Representative Board should consider the advisability of urging the Government to make suitable provisions to that end;

"And (3) that, considering the compulsory attention to the teeth of school children would be a national gain, the Representative Board should be empowered to further the matter in any way they deem most fit."

The resolution was adopted.

During the afternoon a number of demonstrations were given at the Dental Hospital, George Square.

Mr. Kirby exhibited an electric mallet and engine. The apparatus was supplied with current from a set of secondary batteries manufactured and charged by Messrs. Muir, Mavor and Coulson, electric light engineers, Glasgow. These cells require almost no attention further than recharging when run down.

The Hastie motor was shown in work.

Mr. Gordon Jones described a method of treating alveolar abscess.

Mr. Howarth showed the working of his articulator.

Mr. E. Lloyd Williams filled a cavity in a lower molar.

Mr. Coxon exhibited a series of regulation cases, and also a means of shutting off the gas of the Vulcanizer.

Messrs. Ash & Son and the Dental Manufacturing Co. had each a display of instruments and apparatus.

In the evening the annual dinner of the Association was held in the Grand Hotel, Charing Cross. Mr. J. R. Brownlie, President, occupied the chair, and Mr. J. S. Woodburn and Mr. Rees Price acted as croupiers.

The Lord Provost of Glasgow, the President of the Faculty of Physicians and Surgeons, and other distinguished visitors were present, and there was also a large attendance of the members of the Association.

Saturday, the 20th, was devoted to pleasure. A large company visited the shipbuilding yard of Messrs. Denny, at Dumbarton, where, before leaving, a most hospitable repast was provided by the firm. After viewing the works the members of the Association and their friends were entertained by the Scottish Branch. A sail by steamer down the Clyde to the head of Loch Long and back, with a good luncheon on board, terminated another successful meeting of this representative Association.—*London Dental Record.*

